# Early-Stage Evaluation of a Novel Sexual Health Care Model for Breast Cancer Survivors Undergoing Endocrine Therapy: A Case Series

**DOI:** 10.3390/nursrep16090324

**Published:** 2026-09-06

**Authors:** Wakako Yachi

**Affiliations:** Faculty of Nursing, Iwate Prefectural University, 152-52 Sugo, Takizawa 020-0693, Japan; wakako_y@iwate-pu.ac.jp

**Keywords:** breast cancer survivors, endocrine therapy, sexual health, phenomenology, comfort theory

## Abstract

**Background/Objectives:** Women undergoing endocrine therapy after breast cancer often experience sexual-health concerns, but opportunities for individualized nursing support are limited. This case series implemented and preliminarily evaluated a novel sexual health-care model for women undergoing endocrine therapy after breast-cancer treatment. **Methods:** A care model was developed from Comfort Theory, a literature review, and clinical experience and underwent content-validity review by experts, including one nurse with lived experience of breast cancer. It was implemented individually for 6 months in five premenopausal women receiving endocrine therapy after breast-conserving surgery or total mastectomy. Data included baseline characteristics, care records, final interviews, and Self-affirmation Scale scores. Nursing goal attainment and final interviews were evaluated qualitatively. **Results:** All nursing goals appeared to have been achieved based on provider assessment and participants’ narratives. Participants gradually expressed distress, questions, and sexual health needs related to bodily and sexual changes, developed greater understanding of treatment-related changes, identified individualized coping strategies, and showed greater self-worth and self-care. Self-affirmation scores were descriptively higher after care. Phenomenological analysis identified four constituents: having a place to discuss a body marked by breast and fertility loss; sharing with a midwife who accepted them as they were; reconnecting with their changed bodies; and discovering new meaning in life with altered fertility and bodily integrity. **Conclusions:** The care model may provide a safe, individualized space for women undergoing endocrine therapy after breast cancer to express concerns, understand bodily changes, and reconstruct meaning. The findings are preliminary and represent provider-assessed observations rather than evidence of effectiveness.

## 1. Introduction

Women surviving breast cancer experience a wide range of treatment-related sexual health concerns. In particular, women undergoing endocrine therapy (ET) have reported vaginal dryness, dyspareunia, and psychosocial distress related to appearance, body image, and sexuality [1,2]. Sexual dysfunction and related sexual health concerns are common among women receiving adjuvant endocrine therapy following breast cancer treatment. In a prospective cohort of women with early-stage hormone receptor-positive breast cancer receiving adjuvant endocrine therapy, 55.0% reported at least one problem with sexual function, and 45.7% experienced clinically significant deterioration in sexual function [3]. These findings underscore that sexual health concerns during endocrine therapy are neither rare nor incidental but represent clinically important issues warranting systematic assessment and appropriate supportive care. In Japan, Nishio and Sakuda [4] reported that young women with breast cancer desired individualized information and support regarding sexuality during ET. However, nurses often have limited opportunities to address these concerns because hospital stays are short, outpatient follow-up is brief, and nursing care tends to focus on postoperative recovery and treatment management rather than on sexual or menopausal symptoms [5,6].

International oncology and survivorship guidelines likewise emphasize the importance of systematically assessing and addressing sexual health concerns following cancer and breast cancer treatment. The ASCO guideline recommends that a member of the healthcare team initiate discussions with patients regarding sexual health and dysfunction associated with cancer or its treatment [7]. Similarly, the NCCN Survivorship Guidelines provide recommendations for the screening, evaluation, and management of female sexual dysfunction among cancer survivors [8]. Collectively, these recommendations highlight the need for nursing approaches that facilitate open discussion of sexual health concerns and provide appropriate support for women managing these concerns during endocrine therapy.

Sexuality is an important aspect of quality of life for breast cancer survivors. However, women undergoing ET may have limited access to a safe, private environment in which they can discuss sexuality-related concerns with health care professionals. Breast cancer survivors receiving ET often transition quickly from inpatient treatment to outpatient follow-up, and opportunities for continuous nursing support may decrease at a time when women are beginning to experience changes in menstruation, fertility, vaginal symptoms, and emotional responses to treatment. As a result, sexuality-related concerns may remain unspoken, especially when women perceive them as private, embarrassing, or difficult to discuss in routine oncology care. This gap highlights the need for a care approach that not only provides information but also creates a relational context in which women can safely express concerns, make sense of bodily changes, and identify coping strategies in their daily lives.

Although previous studies have described sexual health problems and support needs among women with breast cancer [1,4], little is known about how a structured sexual health care model can be implemented and evaluated in nursing practice for women undergoing ET. The Permission, Limited Information, Specific Suggestions, and Intensive Therapy (PLISSIT) model has been widely used as a framework for addressing sexual concerns in clinical practice [9,10]. Although the PLISSIT model provides an important framework for opening discussion about sexual concerns, the experiences of women undergoing ET after breast cancer extend beyond the need for permission or limited information alone. Their concerns often involve ongoing uncertainty about fertility, altered bodily identity, emotional isolation, and the need to reconstruct meaning in life while living with a changed body. Yachi [11] suggested that comfort in patients with breast cancer includes restoration of confidence in femininity and womanhood, acceptance of one’s situation, identification of coping strategies, and recovery of a sense of control. Taken together, these findings indicate that support for breast cancer survivors undergoing endocrine therapy remains limited and should address not only physical symptoms but also patients’ understanding of their bodily changes, coping strategies, and sense of self.

Comfort Theory was selected as the theoretical foundation because it conceptualizes comfort as a multidimensional experience encompassing relief, ease, and transcendence. Although the biopsychosocial model and Roy’s Adaptation Model provide useful perspectives for understanding the multidimensional effects of illness and processes of adaptation, Comfort Theory was considered particularly well suited to the aims of this study. Specifically, the care model sought not only to address treatment-related symptoms but also to support women in making sense of changes in their bodies, sexuality, fertility-related concerns, relationships, and self-worth during endocrine therapy. This perspective is consistent with previous research on comfort among patients with breast cancer, which suggests that comfort encompasses the restoration of confidence in femininity and womanhood, acceptance of one’s circumstances, identification of coping strategies, and recovery of a sense of control [11].

In the present care model, Comfort Theory was operationalized through four hierarchical nursing goals and four dimensions of comfort. The nursing goals represented progressive care objectives, whereas the four dimensions of comfort—physical, psychospiritual, sociocultural, and environmental—provided a structured framework for assessing participants’ concerns and tailoring the content, communication strategies, and timing of care to individual needs.

Based on these perspectives and using Comfort Theory as the theoretical foundation [12], we developed a sexual health care model to guide individualized support for women undergoing ET after breast cancer treatment. Accordingly, this study aimed to conduct an early-stage case series evaluation of the implementation, acceptability, and perceived utility of a novel sexual health care model for women undergoing endocrine therapy after breast cancer treatment.

## 2. Methods

This was an early-stage, single-group case series using mixed methods to support the preliminary development, implementation, and evaluation of a novel nursing care model. The study was exploratory in nature and was not designed to establish treatment efficacy.

### 2.1. Development of the Care Model

A provisional care model was developed prior to implementation based on Comfort Theory [12], a review of the literature, the author’s clinical experience, and previous research on comfort among patients with breast cancer [11]. The provisional model comprised the conceptual framework, specific care content and evaluation perspectives corresponding to each nursing goal, and the Sexual Health Assessment Sheet. Its content validity and safety were reviewed by one breast specialist, three certified breast cancer nurses, and one nurse with lived experience of breast cancer. The review resulted in no major changes to the model’s core structure, including its four nursing goals and theoretical foundation in Comfort Theory. One practical recommendation was to provide care in a private room to ensure a safe environment for discussing sensitive topics, including sexuality, fertility, bodily changes, and experiences of loss. This recommendation was consistent with the model’s existing emphasis on privacy and a comfortable environment and was incorporated into the specific care strategies.

**Figure 1:**
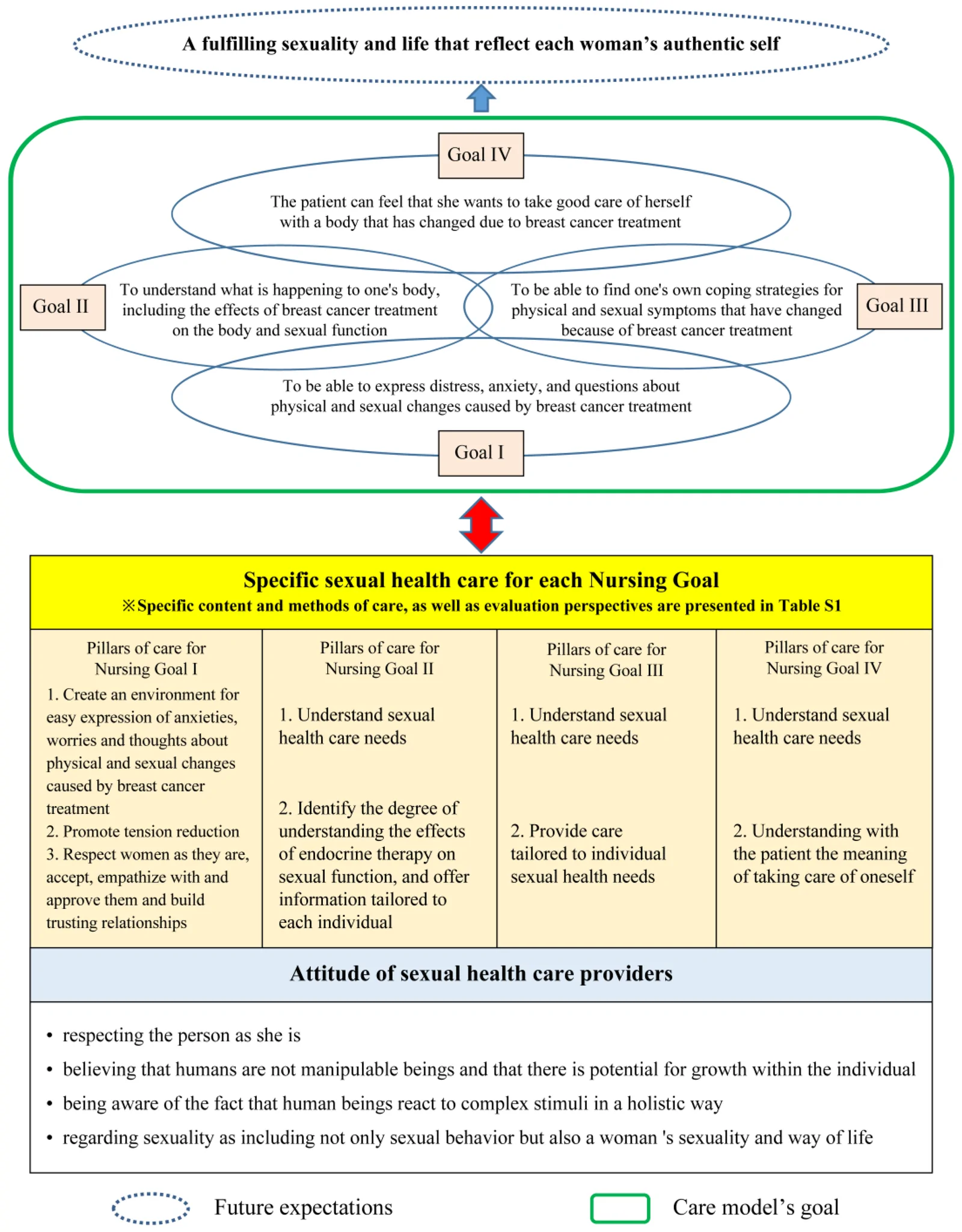
Conceptual model of sexual health care for women with breast cancer undergoing endocrine therapy. The arrows indicate the conceptual progression from specific care to nursing goals and future expectations. Colors are used only to improve readability and visual clarity.

The provisional model was subsequently implemented with five participants. Following completion of the care and final interviews, the model was refined and presented as the final care model shown in Figure 1. At this stage, the hierarchical and overlapping relationships among the four nursing goals were more explicitly represented in the conceptual figure. In addition, the four phenomenological constituents identified through analysis of the final interviews were incorporated into Table S1 to illustrate how participants experienced the care provided within the model. The specific care content and evaluation perspectives were not substantially modified following implementation and are detailed in Table S1. These care components were not intended to constitute a rigid protocol applied uniformly across participants; rather, they served as a flexible framework within which care was individualized according to each participant’s condition, concerns, interests, and responses.

### 2.2. Nursing Goals and Care Content

The model consisted of four hierarchical nursing goals: (1) expressing distress, anxiety, and questions about physical and sexual changes caused by breast cancer treatment; (2) understanding treatment-related bodily and sexual changes; (3) identifying individualized coping strategies; and (4) developing a desire to care for oneself despite these changes. These goals were intended to function sequentially while remaining interactive. In other words, the ability to express distress and questions was considered a prerequisite for a deeper understanding of bodily changes, whereas understanding these changes was expected to facilitate the identification of individualized coping strategies. The final goal was positioned as an outcome reflecting not only symptom management but also a shift in self-perception and self-worth. Care content was based on breast cancer treatment guidelines and was designed not to interfere with treatment.

### 2.3. Care Provider’s Attitude

Because sexuality-related concerns are private and highly individualized, the care model emphasized a nonjudgmental, accepting attitude that respected each participant’s perception of bodily change, fertility, and loss. The care model was reviewed by one breast specialist, three certified breast cancer nurses, and one nurse with lived experience of breast cancer. The nurse with lived experience contributed feedback from both professional and experiential perspectives, particularly regarding the acceptability, sensitivity, and safety of discussing sexuality, fertility, bodily changes, and loss after breast cancer treatment. This feedback informed minor refinements to the care content and communication approach. However, people with lived experience were not formally involved as co-design partners throughout the initial model-development process.

### 2.4. Implementation of Care

Care was provided individually and continuously by a midwife over approximately 6 months, with repeated sessions tailored to each participant’s needs and responses. A Sexual Health Assessment Sheet developed by the researcher was used to assess current symptoms and concerns, as well as relevant surgical procedures and medications (Appendix A). Individual adaptations to care were documented and monitored using the Sexual Health Assessment Sheet, records of each care encounter, field notes, and final interview data. At each session, the researcher assessed participants’ current symptoms, concerns, questions, interests, and responses in relation to the four nursing goals. The content, communication strategies, and timing of care were adjusted according to each participant’s condition and needs while remaining within the overall framework of the care model.

Before and after each session, the researcher reviewed previous assessment information and care records to monitor changes in participants’ concerns, understanding, coping strategies, and expressions of self-care. Care proceeded in accordance with the nursing goals, with the endpoint defined as the apparent attainment of nursing goal IV based on the care provider’s assessment and participants’ narratives. As attainment of the nursing goals was not independently assessed, these endpoint determinations were interpreted as preliminary, provider-assessed indicators rather than independently verified outcomes.

### 2.5. Participants and Recruitment

Participants were recruited from a single cooperating facility using purposive sampling through physician referral. Because the researcher was not employed at the cooperating facility, she was unable to independently identify patients who appeared to meet the study eligibility criteria. Physicians at the facility therefore identified potentially eligible women and referred them to the researcher. The researcher did not have access to information regarding the total number of women initially considered or approached by the physicians. The researcher provided a direct explanation of the study to the five women referred by the physicians, invited them to participate, and obtained written informed consent. All five women agreed to participate, and none subsequently withdrew or discontinued participation.

The eligibility criteria were women aged 50 years or younger who had been diagnosed with primary breast cancer, had undergone breast-conserving surgery or total mastectomy, were premenopausal, were receiving endocrine therapy after breast cancer treatment, were able to communicate verbally, and provided written informed consent to participate. Participants were also required to be able to participate in repeated individualized care sessions and interviews over approximately 6 months. Participants were between the fourth day and the second month after initiation of endocrine therapy at the start of care.

### 2.6. Reflexivity and Researcher Characteristics

The author was a female midwife and nursing researcher with clinical experience in women’s health, midwifery, and breast cancer-related sexual health care. She developed the care model, recruited participants, delivered the intervention, assessed nursing goal attainment, and conducted the primary qualitative analysis.

The author was not employed by the hospital at which participants received treatment, had no prior relationship with the participants before recruitment, and was not involved in their routine oncology care or treatment decisions. Participants were informed of the author’s role as a midwife and researcher conducting the study, that participation was voluntary, and that refusal to participate or subsequent withdrawal would not affect their medical treatment or care at the hospital.

As the author served as both the care provider and interviewer, participants may have been inclined to provide more favorable accounts of their experiences, introducing the possibility of social desirability bias. Furthermore, the author’s involvement in both developing and delivering the care model may have influenced the assessment of nursing goal attainment and the interpretation of the qualitative data. To enhance reflexivity and mitigate these potential influences, the author repeatedly returned to the original interview transcripts, care records, and field notes throughout the analytic process and sought to bracket assumptions arising from her professional background as a midwife and nursing researcher. The analysis was conducted in consultation with a qualitative researcher with expertise in phenomenology.

### 2.7. Data Collection

Data included baseline characteristics, the Sexual Health Assessment Sheet, records of each care encounter, field notes, final interview transcripts, and the Self-affirmation Scale [13]. The Self-affirmation Scale was administered twice, at the initial care session and at the final interview.

The Self-affirmation Scale Ver. 2 assesses attitudes and feelings related to individuals’ positive perceptions and evaluations of themselves. The scale comprises eight items, including four reverse-scored items, each rated on a 4-point scale. Total scores range from 8 to 32, with higher scores indicating greater self-affirmation. The reliability and validity of the scale have been examined in previous studies [13]. Permission to use the scale was obtained from the developer prior to the study. Given the small sample size of this case series, scores on the Self-affirmation Scale Ver. 2 were treated as supplementary descriptive quantitative data to characterize changes observed over the course of care and were not used for inferential statistical analysis.

### 2.8. Evaluation of the Care Model

The care model was evaluated qualitatively by examining the apparent attainment of nursing goals, as assessed by the care provider based on observed changes in participants’ narratives and behaviors during the course of care. Because these assessments were not independently verified, they were treated as preliminary, provider-assessed indicators rather than objective evidence of effectiveness. The model was evaluated from the participants’ perspectives through qualitative analysis of their experiences of receiving the care. Changes in Self-affirmation Scale scores from before to after care were used as supplementary descriptive quantitative data.

### 2.9. Data Analysis

Nursing goal attainment was assessed through qualitative analysis of changes in sexual health status based on the Sexual Health Assessment Sheet, care records, field notes, and interview data. Verbatim transcripts were prepared from the recorded care sessions and final interviews. Interactions between participants and the care provider, including silences, pauses, nursing approaches, and behavioral changes, were examined qualitatively.

Final interview transcripts were analyzed with reference to Giorgi’s descriptive phenomenological method [14]. First, each transcript was read repeatedly to develop a sense of the whole. Second, meaning units were identified at points where shifts in meaning relevant to the research aim occurred. Third, these meaning units were transformed from participants’ everyday expressions into phenomenologically sensitive descriptions while remaining grounded in the original data. Fourth, the transformed meaning units were compared across the data and synthesized into phenomenological constituents. Finally, the constituents were integrated to describe the overall structure of participants’ experiences of receiving care within the sexual health care model.

Trustworthiness was addressed with reference to Lincoln and Guba [15] through prolonged engagement, verbatim transcription, transparent documentation of the analytic process, and review by experts in maternal nursing and phenomenology.

Self-affirmation Scale total scores were calculated for each participant before care and after the final interview, and the scores were compared to supplement the qualitative findings.

### 2.10. Ethical Considerations

This study was conducted in accordance with the principles of the Declaration of Helsinki. Participants were informed of the purpose and methods of the study, the voluntary nature of participation, their right to withdraw at any time, and the protection of personal information. Written informed consent was obtained from all participants. If severe psychological distress or an urgent need for support was identified during the study, the researcher planned to consult and collaborate with the participant’s treating physician at the cooperating facility. This response plan was discussed with the physician in advance, and arrangements were made for appropriate clinical support to be provided if necessary.

To ensure the independence of the consent process and minimize any perceived pressure or influence from treating physicians, the physicians were not involved in providing the detailed study explanation or obtaining informed consent. The author had no prior relationship with the participants before recruitment and was not involved in their routine oncology care or treatment decisions. Participants were explicitly informed that declining participation or subsequently withdrawing from the study would not affect their medical treatment or care at the hospital.

Participants were also informed that care would be discontinued if the process caused distress and that collaboration with a breast specialist would be arranged if necessary. The study was approved by the Iwate Prefectural University Graduate School of Nursing Research Ethics Review Committee (2018-D001, 2019-D002).

## 3. Results

### 3.1. Characteristics of the Participants

Five women participated in the study. Their background characteristics are presented in Table 1. The mean age of the participants was 41.2 ± 4.9 years. All participants were employed and premenopausal. Three had undergone breast-conserving surgery, and two had undergone total mastectomy. All were receiving ET, and some had also received radiation therapy or luteinizing hormone-releasing hormone agonist treatment.

### 3.2. Implementation of the Care Model

The duration of care was approximately 6 months. The interval between sessions was determined through agreement between the participants and the researcher and ranged from approximately 1–2 months. The total duration of care was 1675 min, and the mean duration per session was approximately 59 min. Ms. A received care six times, Ms. B five times, Ms. C four times, Ms. D four times, and Ms. E five times. All sessions were conducted in a private room to ensure privacy and facilitate discussion of sensitive concerns.

### 3.3. Achievement of Nursing Goals Through the Sexual Health Care Model

During implementation of the sexual health care model, the four nursing goals appeared to have been attained based on the provider’s assessment and participants’ narratives.

#### 3.3.1. Evaluation of Nursing Objective I

Participants perceived breast cancer and related sexual health concerns as highly private and difficult to discuss openly. A private one-to-one setting with a midwife enabled them to speak about these concerns more calmly and safely. As one participant stated, *“Breast cancer, cervical cancer, and other women’s diseases are sometimes difficult to talk about openly, so it is a sensitive part” (Ms. B).* Another said, *“I feel very hurt and pained when someone talks about it openly, so if we have a chance to talk about it in a private room like this, I can talk calmly and feel comfortable” (Ms. D).*

Through repeated care, all five participants gradually expressed distress, anxiety, doubts, and hopes regarding the physical, mental, and sexual changes caused by breast cancer treatment. Some participants cried while describing experiences such as total mastectomy and their inability to accept the wound. They were also able to talk about menstruation, fertility, and the meanings these experiences held for them. For example, menstruation represented the possibility of future pregnancy for one participant and womanhood for another. Although sexual health needs differed among participants, all five women became able to express concerns related to breast cancer treatment in a calm, private setting. Thus, the nursing goal I appeared to have been attained based on the provider’s assessment and participants’ narratives.

#### 3.3.2. Evaluation of Nursing Objective II

All participants showed interest in understanding the changes occurring in their bodies, including the effects of breast cancer treatment on sexual and reproductive function. They asked questions about menstruation, hormonal side effects, vaginal symptoms, fertility, and contraception. For example, one participant asked, *“How long do the side effects of hormonal agents take, and do some people not get their periods back?” (Ms. A)*, while another said, *“I don’t have my period, so I wonder if that is a side effect or not” (Ms. A)*.

When the researcher provided explanations tailored to each participant’s concerns, participants showed greater understanding of their bodies and treatment-related changes. They also expressed interest in practical aspects of bodily care, such as genital hygiene, menstrual changes, and the implications of hormone therapy for pregnancy. One participant commented, *“If I can take care of myself regularly now that I have breast cancer, I think it would be beneficial in terms of knowing my own body” (Ms. E)*. These findings suggest that all participants became interested in understanding their bodily changes and were able to receive and use individualized information. Thus, nursing goal II appeared to have been attained based on the provider’s assessment and participants’ narratives.

#### 3.3.3. Evaluation of Nursing Objective III

All participants were able to express sexual health needs related to breast cancer treatment, including distress, doubts, wishes, and hopes, and to consider coping strategies suited to their own situations. Their concerns included altered body image, corrective underwear, clothing choices, fertility, sexual and reproductive uncertainty, and future treatment decisions. For example, one participant said, *“I want to wear more beautiful and fashionable underwear, and what kind of underwear should I buy?” (Ms. B)*.

Participants attempted to cope with their changed bodies in their own ways, based on their own knowledge and experience. When knowledge gaps were identified, the researcher provided individualized professional information to supplement their understanding and support their decision-making. In this way, participants were able to identify or strengthen coping strategies appropriate to their own circumstances. Thus, nursing goal III appeared to have been attained based on the provider’s assessment and participants’ narratives.

#### 3.3.4. Evaluation of Nursing Objective IV

Over time, participants began to express greater acceptance of their changed bodies and a stronger desire to care for themselves. For example, one participant initially spoke with confusion about losing the future she had imagined and asked how she should come to terms with that loss. By the third care session, however, she said, *“I hope I can control the side effects as little as possible so that we can get along well with each other” (Ms. D)*, indicating a shift toward acceptance and self-management. She also stated, *“I have to do what I think now” (Ms. D)*, reflecting a changed perspective on life and greater self-worth.

Other participants also described future-oriented and self-affirming attitudes, such as wanting to continue treatment, live in a healthy way, engage in activities they enjoyed, or encourage others to undergo screening. Although the degree of self-acceptance varied among participants, all showed behaviors or statements indicating that they valued themselves and wished to care for themselves. The care sessions also helped them identify what was important in their lives and express this more clearly.

Thus, nursing goal IV appeared to have been attained based on the provider’s assessment and participants’ narratives. No participants were identified as having significant psychological distress requiring urgent psychological or psychiatric referral during the care period or final interview. Therefore, no participant was referred to a clinical psychologist, psychiatrist, or psycho-oncology service during the study.

### 3.4. Phenomenological Findings: Constituents and Overall Structure of Participants’ Experiences

Phenomenological analysis of the final interviews identified four constituents characterizing the lived experiences of women who received this sexual health care. These constituents were integrated into an overall experiential structure, indicating that participants experienced the care not merely as the provision of information but as a relational and embodied process through which they could express distress, reconnect with their bodies, and reconstruct meaning in their lives.

Constituent 1: “A place where I can talk as much as I want about myself, a body that has lost its breasts and fertility and has been damaged.”

Participants described their breast cancer experiences as difficult to share with others, particularly because the loss of breasts, fertility, and bodily familiarity was closely tied to their sense of womanhood and imagined future. They often felt lonely, misunderstood, and unable to speak openly, even to those close to them. In this context, the care setting became a place where they could speak freely and calmly about their changed and damaged bodies.

*“It was really helpful to be able to hear various kinds of information, and because I rarely have opportunities to talk about my cancer, being able to speak about what I have been thinking feels very relieving.”*

(Ms. A)

Constituent 2: “Sharing with a midwife who accepts ‘me’ as I am.”

Participants experienced the midwife as someone who listened without judgment or denial and accepted their suffering as it was. This acceptance enabled them to share experiences that had remained unspoken, including pain associated with bodily loss, disrupted life plans, and feelings of isolation. Through this relationship, the women felt that their suffering was acknowledged and shared.

*“I think it was because I had someone who listened to me like this and accepted what I said that I was finally able to begin thinking through many things within myself.”*

(Ms. D)

Constituent 3: “Turning the gaze on the entangled body and recovering the voice of the body.”

Through dialogue with the midwife, participants gradually turned their attention toward bodies they had previously experienced as damaged, distant, or difficult to face. They came to reflect on menstruation, fertility, genital symptoms, and bodily sensations with greater awareness and understanding. Professional explanations from the midwife helped them make sense of these experiences and supported a renewed ability to listen to and care for their own bodies.

*“Before, my periods used to come very regularly, every 30 days. I kept track of them on my phone, but after the surgery, they started coming in a pattern of 30 days, then 40 days, then 30, then 40.”*

(Ms. B)

Constituent 4: “Discovering new meaning in one’s existence through living in a body that cannot give birth, a body that can give birth, and a woman’s body.”

Participants described a gradual process of reinterpreting their changed bodies and lives. Although breast cancer treatment had altered their fertility, bodies, and future expectations, the care helped them reconsider what it meant to live as women and to value themselves. Through this process, they began to discover new meaning in their lives and existence.

*“I do sometimes think that if I stopped treatment, maybe I could still have a child in about 6 months. Part of me still wonders whether it might not be too late. But at the same time, if I stopped treatment and then the cancer recurred and my life became shorter, I wonder whether that would make the child unhappy.”*

(Ms. D)

### 3.5. Evaluation of the Self-Affirmation Scale Ver. 2

Individual pre- and post-care Self-affirmation Scale Ver. 2 scores and corresponding change scores are presented in Table S2. Self-affirmation scores increased in all participants following care, with individual changes ranging from +4 to +7 points. The mean score increased from 20.4 before care to 25.2 after care. Given the small sample size and descriptive nature of this case series, these scores were used solely as supplementary quantitative data to characterize changes observed over the course of care and to contextualize the qualitative findings. Accordingly, these descriptive changes should not be interpreted as evidence of treatment efficacy or statistically demonstrated effectiveness.

## 4. Discussion

Women undergoing ET after breast cancer often experience sexual health concerns related not only to physical symptoms but also to fertility, body image, self-worth, and relationships. Previous studies have shown that sexual health support for breast cancer survivors remains insufficient, despite patients’ preferences for communication with health care professionals about these issues [1,16,17,18]. The present early-stage case series suggests that the sexual health care model provided a safe, private space in which women could express concerns that had previously remained unspoken, understand changes in their bodies, identify individualized coping strategies, and gradually regain a sense of self-worth.

### 4.1. Evaluation of the Care Model for Women Undergoing ET

The participants in this evaluation study were all premenopausal women in the early phase of ET. This period is clinically important because patients may begin to experience treatment-related bodily changes and uncertainty while receiving limited nursing support during outpatient follow-up [19]. In this study, participants did not necessarily present with severe menopausal or genital symptoms, but they did express concerns about menstruation, fertility, anxiety, irritability, bodily changes, and the future. These findings suggest that support in the early phase of ET should address not only severe physical symptoms but also emotional distress, uncertainty, and sexual health needs that may be difficult to express in routine clinical settings.

The four nursing goals appeared to function hierarchically. Participants first needed a safe setting in which they could express distress, anxiety, and questions regarding bodily and sexual changes. Once this foundation was established, they were better able to understand treatment-related changes, consider ways of coping, and eventually express a desire to care for themselves. This progression suggests that the first nursing goal, enabling women to talk openly about private and sensitive experiences, provided the foundation for achieving the subsequent goals.

The findings of this initial evaluation study also indicate that the care model was particularly meaningful for women who perceived breast cancer as a deeply private and gendered experience. Participants experienced the loss not only of breasts but also of fertility, bodily familiarity, and aspects of the future they had imagined for themselves. These experiences were difficult to share with family members or others close to them. The one-to-one care sessions, therefore, functioned as a space where participants could speak honestly about their changed bodies, loss, and uncertainty without fear of being hurt, dismissed, or misunderstood.

### 4.2. Significance of Midwife-Led Sexual Health Care

A notable feature of this evaluation study was that the care was provided by a midwife. Participants perceived the midwife as a professional who could address topics such as menstruation, fertility, vaginal symptoms, sexuality, and reproduction openly and knowledgeably. This may have reduced embarrassment and encouraged women to discuss issues often left unaddressed in cancer care. The findings suggest that midwife-led care may be especially valuable for women whose concerns involve fertility, menstruation, intimate body changes, and their identity as women.

The care provider’s approach appeared central to participants’ experiences of the model. Care was characterized by acceptance, non-judgment, and respect for each participant’s individual experience. Participants described difficulty sharing their experiences with others and, in some cases, feeling hurt by others’ responses. By contrast, the midwife’s acceptance of participants’ narratives without judgment appeared to facilitate self-expression, self-acceptance, and reconnection with their bodies. Thus, the perceived value of the model may derive not only from the information provided but also from the relational and emotional qualities of the care.

The phenomenological constituents derived from the final interviews further deepen the interpretation of these findings. Participants did not experience the care merely as a source of information or advice; rather, they experienced it as a place where they could talk about bodies marked by the loss of breasts and fertility, share their suffering with a midwife who accepted them as they were, reconnect with their changed bodies, and begin to reconstruct meaning in their lives. These findings suggest that sexual health care for women undergoing ET after breast cancer should be understood broadly, not only as support for symptom management or coping with sexual dysfunction but also as support for embodied and existential recovery, including bodily meaning, identity, and self-worth.

The phenomenological findings also suggest that the significance of sexual health care in this population lies in its continuity and relational depth. Through repeated encounters, participants were able to move from silence and uncertainty toward expression, understanding, and self-care. This process may be especially important for women in the early phase of ET, when distress is present but may not yet be articulated as a clinical problem. Previous studies have shown that women with breast cancer value opportunities for patient–provider communication about sexual concerns and that sexual health problems may persist over time and remain closely related to quality of life and treatment burden [1,4,20]. In addition, qualitative work has highlighted that breast cancer-related bodily changes are experienced not only physically but also existentially, affecting women’s sense of self and everyday life [21]. In this sense, sexual health care may function not only as supportive counseling but also as a form of nursing care that helps women reinterpret bodily changes and sustain a sense of self in everyday life.

The findings can also be interpreted in light of Comfort Theory. The care model supported not only relief from distress but also a broader sense of ease through acceptance, understanding, and regained agency. In this sense, comfort was not limited to symptom reduction; it also included participants’ ability to live with changed bodies while recovering a sense of femininity, continuity, and self-value.

### 4.3. Appropriateness of the Nursing Goals and Specific Care

The four nursing goals used in this study appear to have been appropriate for this population. Goal I, which focused on the expression of distress, anxiety, and questions, was essential because sexual health issues were experienced as sensitive, private, and difficult to discuss. Goals II and III, understanding bodily changes and identifying individualized coping strategies, were also appropriate because participants needed both specialized knowledge and support to make sense of their own bodily experiences. Goal IV, developing a desire to care for oneself, captured an important outcome of care that extended beyond symptom management.

The increases in self-affirmation scores following care were consistent with the qualitative findings and were interpreted as supplementary descriptive indicators of changes in participants’ self-worth and self-regard. Self-affirmation reflects positive and favorable attitudes toward oneself [13], and the observed increases may therefore reflect greater self-worth and positive self-regard over the course of care. This interpretation was consistent with the qualitative findings, in which participants gradually expressed greater self-acceptance, hope, and concern for their own well-being.

### 4.4. Implications for Nursing Practice

The findings of this study suggest that women undergoing ET after breast cancer need opportunities to discuss sexuality, fertility, menstruation, bodily changes, and self-care in a safe, private environment. Such opportunities are difficult to secure in routine outpatient practice, where contact with nurses may be limited. The present care model may therefore offer a practical framework for providing individualized sexual health support to breast cancer survivors undergoing endocrine therapy.

The study also suggests that sexual health care should not be reduced to the management of vaginal dryness or menopausal symptoms alone. Rather, care should be based on a broader understanding of women’s lived experiences, including loss, uncertainty, femininity, identity, and the desire to continue living meaningfully in a changed body. Strengthening education and training in sexual health care and women’s health for nurses, certified breast cancer nurses, oncology nurses, and midwives may help expand such care in clinical practice.

### 4.5. Limitations

This study has some limitations. First, a major methodological limitation was the single-provider, single-analyst structure, whereby the same midwife served as the model developer, care provider, evaluator of nursing goal attainment, interviewer, and primary qualitative analyst. This structure may have introduced expectation, confirmation, and observer biases, as well as social desirability bias. In particular, nursing goal attainment was assessed by the care provider rather than by an independent evaluator; consequently, findings regarding goal attainment should be interpreted as preliminary, provider-assessed observations rather than independently verified outcomes. Although the author repeatedly returned to the original transcripts and care records and sought to bracket assumptions arising from her professional background, interpretation of participants’ experiences may nevertheless have been influenced by her expectations regarding the care model and her involvement in delivering the intervention. Furthermore, although the analytic process was conducted in consultation with a qualitative researcher with expertise in phenomenology, neither independent analysis of a subset of the data nor independent assessment of nursing goal attainment was conducted.

Second, the supplementary quantitative evaluation was limited to the Self-affirmation Scale Ver. 2. Although these scores were used solely as descriptive data to complement the qualitative findings, the study did not include additional validated measures of sexual functioning, body image, psychological distress, menopausal symptoms, or quality of life. Consequently, changes across multiple dimensions of participants’ sexual health and psychosocial well-being could not be quantitatively assessed.

Third, although the small sample was suitable for an in-depth phenomenological exploration, the study was a single-group case series involving only five participants recruited from a single institution. Participants shared relatively similar clinical contexts, further limiting the transferability of the findings. Moreover, in the absence of a comparison or control group, changes observed over the course of care could not be distinguished from natural adaptation over time during endocrine therapy or from other contextual influences. The findings, therefore, should not be interpreted as evidence of a causal effect of the care model. Participants were in the early phase of endocrine therapy and did not necessarily experience severe sexual dysfunction; thus, the applicability of the model to women with more severe, persistent, or complex sexual health concerns remains to be established.

Fourth, care was provided over approximately 6 months, and no follow-up assessment was conducted after its completion. The long-term durability and sustainability of the observed changes therefore could not be evaluated. In particular, it remains unclear whether changes in self-affirmation, coping strategies, and self-care would be maintained after completion of the care period.

Finally, patient and public involvement in model development was limited. Although one individual with lived experience of breast cancer, who was also a nurse, participated as a patient expert in the content-validity review and contributed to minor refinements of the model, people with lived experience were not systematically engaged as co-design partners from the outset. Moreover, the lived-experience perspective was represented by a single individual whose professional background in healthcare may not reflect the diversity of experiences, needs, and priorities among breast cancer survivors. Future refinement of the model should therefore involve multiple breast cancer survivors as patient partners throughout the processes of co-design, implementation, evaluation, and dissemination.

### 4.6. Future Directions

Future studies should employ multicenter designs with larger, more diverse, and more representative samples to examine the applicability and transferability of the model across different clinical settings and patient populations. In addition, independent assessment, multidimensional validated outcome measures, and longer-term follow-up after completion of care are needed to evaluate the model more comprehensively and rigorously.

## 5. Conclusions

In the present study, we evaluated the implementation, acceptability, and perceived utility of a sexual health care model delivered over approximately 6 months to five premenopausal women undergoing ET following breast cancer surgery. Through repeated, individualized care, participants gradually expressed distress, questions, and sexual health needs related to treatment-associated bodily and sexual changes. With support tailored to their individual needs and levels of understanding, participants appeared to develop a better understanding of these changes, identify coping strategies, and express greater self-worth and engagement in self-care. All four nursing goals appeared to have been attained based on the care provider’s assessment and participants’ narratives. Phenomenological findings further indicated that participants experienced the care as a safe space in which they could discuss experiences of loss, bodily changes, fertility-related concerns, and sexuality; share their suffering with a midwife who responded with acceptance and without judgment; reconnect with their changed bodies; and begin to reconstruct meaning in their lives.

These preliminary case-series findings suggest that the model may be feasible and acceptable and may have potential utility as a safe, individualized framework for sexual health care. However, given the small sample size, single-group case-series design, absence of a comparison group, reliance on provider-assessed nursing goal attainment, and lack of follow-up assessment, the present study cannot establish the effectiveness or causal effects of the model. Further research incorporating co-design with diverse breast cancer survivors, multicenter studies with larger and more representative samples, independent assessment, multidimensional validated outcome measures, and longer-term follow-up is needed to refine the model and evaluate its feasibility, acceptability, and potential benefits more rigorously.

## Figures and Tables

**Table 1:** Characteristics of the participants.

Participant:	A	B	C	D	E
Age	Early 40s	Late 40s	Early 30s	Early 40s	Early 40s
Surgical method	Breast-conserving surgery	Breast-conserving surgery	Breast-conserving surgery	Total mastectomy	Total mastectomy
Treatment	Radiation therapy Tamoxifen	Radiation therapy Tamoxifen	Radiation therapy Tamoxifen LH-RH agonist	Tamoxifen	Tamoxifen
Employment status	Yes	Yes	Yes	Yes	Yes
Marital status	Married	Unmarried	Unmarried	Unmarried, with a partner	Unmarried
Menstrual status	Premenopausal	Premenopausal	Premenopausal	Premenopausal	Premenopausal

**Figure A1:**
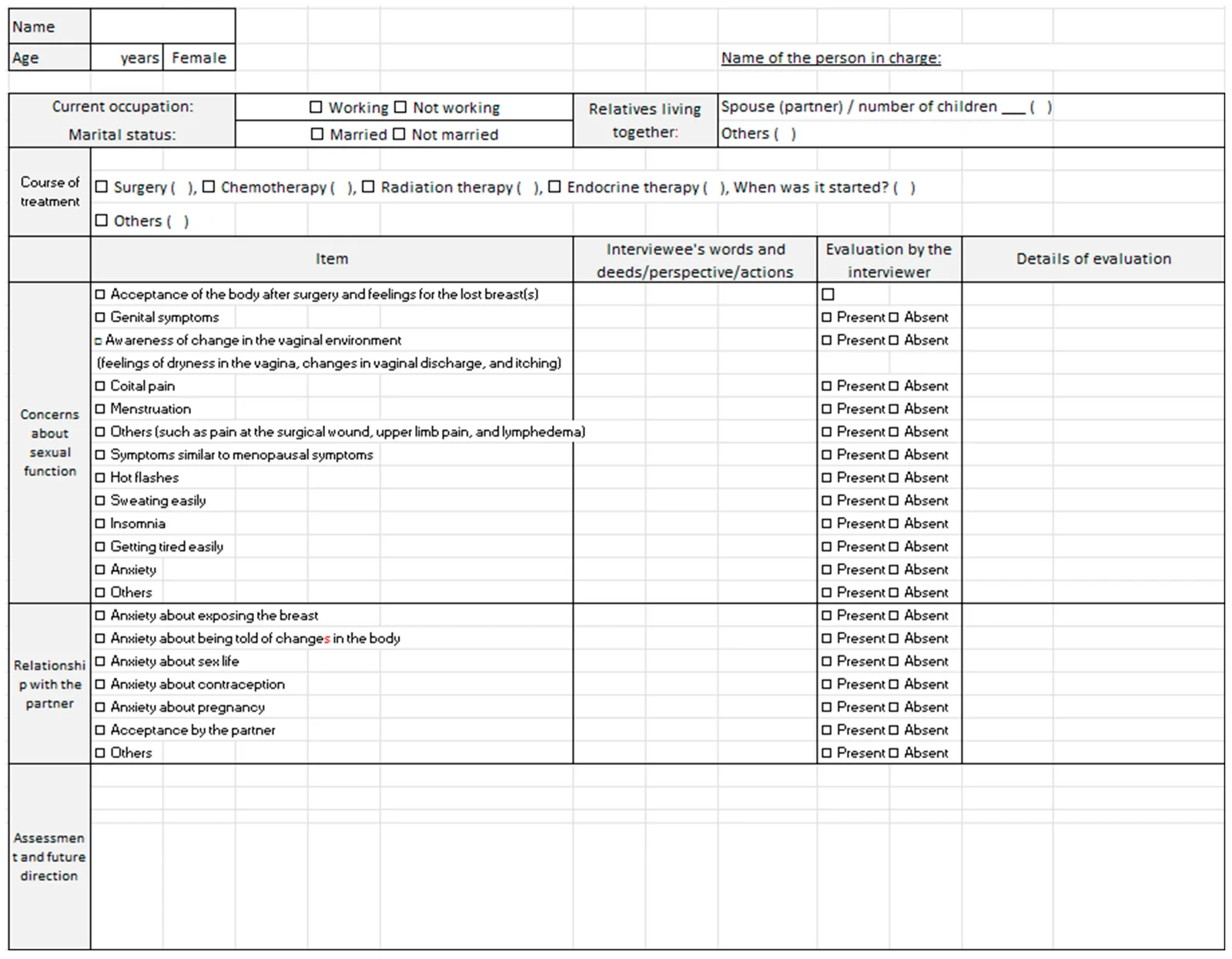
Assessment sheet for the sexual health of women with breast cancer. Assessment was performed on month __ date ___, year ___ [first time/___ time].

## Data Availability

The data presented in this study are available on request from the corresponding author due to the data containing information that could compromise the privacy of research participants.

## Supplementary Materials

The following supporting information can be downloaded at https://www.mdpi.com/article/10.3390/nursrep16090324/s1, Table S1: Overview of the Sexual Health Care Model: Specific content of sexual health care, according to each nursing goal; Table S2: Individual pre- and post-care Self-affirmation Scale Ver. 2 scores and change values.

## Public Involvement Statement

During the content-validity review of the care model, one nurse with lived experience of breast cancer provided feedback from both professional and experiential perspectives. Her feedback informed minor refinements of the care content and communication approach. However, people with lived experience were not formally involved as co-design partners throughout the initial development process, and no study participant was involved in the design, analysis, interpretation, or dissemination of the study findings.

## Guidelines and Standards Statement

The reporting of the qualitative component of this study was guided by the Consolidated Criteria for Reporting Qualitative Research (COREQ) checklist.

## Use of Artificial Intelligence

During the preparation of this manuscript, the author employed the DeepL Translator Pro tool web version (https://www.deepl.com/en/pro, accessed on 18 June 2026), to facilitate the translation of the original Japanese manuscript and revised text into English. The translated manuscript was subsequently subjected to professional English-language editing. The study’s design, execution, analysis, and interpretation were conducted without the utilization of artificial intelligence or AI-assisted tools. The author has reviewed and approved the final manuscript and accepts full responsibility for its content.

## Abbreviations

The following abbreviations are used in this manuscript:

BC	Breast Cancer
ET	Endocrine Therapy
HUNT	Trøndelag Health Study (Helseundersøkelsen i Nord-Trøndelag)
LHRH	Luteinizing Hormone-Releasing Hormone
PLISSIT	Permission, Limited Information, Specific Suggestions, and Intensive Therapy
QOL	Quality of Life
