# Supplementary material for: Early-Stage Evaluation of a Novel Sexual Health Care Model for Breast Cancer Survivors Undergoing Endocrine Therapy: A Case Series
Source: Nurs Rep. 2026 Sep 6;16(9):324. doi: 10.3390/nursrep16090324 (PMC13610420; doi:10.3390/nursrep16090324)
Supplement: Supplementary file 1 [file nursrep-16-00324-s001.zip › Table S1.pdf]

Supplementary Table S1. Overview of the Sexual Health Care Model: Specific content of sexual health care, according to each nursing goal

Nursing goals	Nursing goal I: To ensure the patient is able to express distress and anxiety, and to ask questions about physical and sexual changes caused by the breast cancer treatment	Nursing goal II: To ensure the patients understand what is happening to one's body, including the effects of the breast cancer treatment on the body and sexual function	Nursing goal III: To ensure the patient is able to find her own coping strategies for physical and sexual symptoms that have changed because of the breast cancer treatment	Nursing Goal IV: To ensure the patient can feel that she wants to take good care of herself with a body that has changed due to breast cancer treatment
Evaluation perspectives	・Key words and behavior reveal that she feels relaxed and secure.<br>・In response to the caregiver's questions, she expresses her distress, worries, and doubts about the physical, mental, and sexual changes caused by her breast cancer treatment.<br>・She spontaneously expresses her distress, worries and doubts about the physical, mental, and sexual changes caused by her breast cancer treatment.<br>・She is able to expresses, in particular, her sexual health care needs (distress, doubts, worries, intentions, current situation and hopes) relevant to her breast cancer treatments	・She uses words and actions to indicate that she is focusing on what is happening to her body and the effect of the breast cancer treatment on her sexual function.<br>・She is able to express her sexual health care needs (pain, doubts, distresses, intentions, current situation and hopes) that accompany the specific breast cancer treatments.<br>・She states that she wants to understand what is happening to her body.<br>・She uses words that indicate that she understands her own body, in her own way, including the effects of breast cancer treatment on her body and sexual function.<br>・She expresses positive words and actions about her peace of mind and acceptance of the physical, mental and sexual changes that result from the breast cancer treatment.	・She is able to expresses the sexual health care needs (pain, doubts, distresses, intentions, current situation and hopes) accompanying the specific breast cancer treatment.<br>・She uses words and actions that reveal that she is trying to find her own ways to cope with her sexual health care needs in response to the physical and sexual symptoms caused by breast cancer treatment.<br>・She can consider and choose her own ways of coping with the sexual health care needs that accompany the physical and sexual symptoms resulting from the breast cancer treatment.<br>・If she is already coping in her own way, she is able to look back on, reconsider, and evaluate, her actions.<br>・Even if she is already coping in her own way, she uses words and shows actions that indicate that she is willing to consider better ways, if necessary.	・She uses words and shows actions that indicate that she is accepting and affirming what is happening to her own body, including the effects of the breast cancer treatment on her body and sexual function.<br>・She is expressing her feelings about taking care of herself.<br>・She uses words that reveal that she has become aware of, and is accepting and affirming, her values and beliefs about her own sexuality.<br>・She is taking actions to improve her physical condition, and to heal, please, and relax herself.<br>・She uses words that reveal her trust and confidence in herself.<br>・She is being proactive, such as by undergoing routine medical checkups exams and conducting breast self-examinations.
Specific content and methods of sexual health care	**1. Create an environment for easy expression of anxieties, worries, and thoughts about the physical and sexual changes caused by breast cancer treatment**<br>1) Secure time and space for the participants in places where their privacy can be protected.<br>2) Offer a pleasant physical environment in which the participants can relax as much as possible by using relevant tools, while considering their hopes and preferences.<br>**2. Promote tension reduction**<br>1) For the first several sessions, avoid focusing on one-sided data collection or interrogations, to ease the participants' tension and create an atmosphere in which everyone can talk freely. Try to encourage the participants to take part in the conversations in their own way, when they are ready.<br>2) To ease tension and ensure mental stability, use breathing techniques, hand massage, touch care and warm compresses, in line with the participants' wishes and conditions. Provide advice on ways to relax, as needed, and engage in them together.<br>**3. Respect women as they are. Accept, empathize with, and affirm them to build trusting relationships**<br>1) Understand that the participant has survived breast cancer and is burdened with an unstable condition and must continue to live with the fear of cancer recurrence and death, as well as endure long-term endocrine therapy.<br>2) Do not dismiss, based on a midwife's values, any views or conclusions on breast surgery and treatment that the participants have arrived at on their own, but respect them as they are.<br>3) Use words that positively affirm the efforts, ways of coping, and strengths of the participants. While interacting with the participants, midwives should try to discover, and convey verbally, that, even though they have experienced breast cancer, their attractiveness as a human being and as a woman are unchanged, and will remain so.<br>4) Ask the participants how they themselves feel about their surgical scars, and whether they perceive a sense of loss as a woman owing to the loss of her breasts because of breast surgery, and if so, ask about their reason(s). Since the strength of this feeling and the reason(s) are unique, with some perceiving a sense of loss and others not reacting in this way, accept the situation as is without making any casual judgments or evaluations based on a midwife's set of values, including "Your scar is clean and beautiful," and "Everything is OK; there is nothing to worry about." Subsequently, convey to them that, despite breast deformity and loss, their basic nature and femininity remain intact.<br>5) The midwives themselves should make self-introductions and self-disclosure. They will also convey to the participants that midwifery is an occupation that holds expertise related to women's lifelong health and sexual wellbeing.<br>6) Tell the participants, and ensure they understand, that they are a special presence, and that this is a forum where people can talk about breasts, sex, and menstruation.<br>7) Avoid dismissing the experiences shared by the participants and accept them as they are. Sympathize with and support	**1. Understand sexual health care needs**<br>1) Use the originally-created sexual health assessment sheet to assess the sexuality unique to the person.<br>**2. Identify the patient's degree of understanding the effects of endocrine therapy on sexual function, and offer information tailored to each individual**<br>1) Understand to what extent the participants understand the effects of endocrine therapy on female hormones, sexual function, sexual desire, and menstruation, etc. Proceed while listening carefully to how the patients perceive the changes in their bodies, sex, and menstruation after breast cancer surgery, making sure that this does not become a one-way provision of knowledge and information. Use words and expressions that allow them to perceive the matter positively.<br>2) As vaginal dryness and reduced sexual desire are known effects of hormone therapy, tell them that these symptoms do not indicate that they love their partner less.<br>3) Sometimes, make use of models such as that of the uterus and ovaries according to individual needs, to deepen their understanding of the female anatomy.	**1. Understand sexual health care needs**<br>1) Use the originally-created sexual health assessment sheet to assess the sexuality unique to the person.<br>**2. Provide care tailored to individual sexual health needs**<br>1) If menopausal-like symptoms appear due to endocrine therapy, listen to the participants' thoughts and their strategies to handle them, and consider taking possible countermeasures together. For their own sexual health, propose self-care strategies they are likely able to carry out themselves. Respect the fact that they chose and decided on these on their own.<br>2) If they wish to enjoy fashion in their own way by wearing breast enhancements and corrective underclothing, show what products are available and help them with fittings.<br>3) If they have any symptoms specific to their reproductive system, listen attentively to their thoughts and learn about their coping methods. Acknowledge the participants' efforts in dealing with their symptoms and convey this verbally. If they wish, offer lubricating jelly to counter vaginal dryness, and provide information regarding moisture care.<br>4) Inform them of the need and measures to prevent vaginitis due to a reduction in the vagina's self-cleansing capabilities. Encourage them to avoid, as much as possible, wearing over-tight and unventilated underwear and pants. They should replace panty liners and sanitary napkins every 2-3 hours. When taking a bath, they should not roughly scrub the outer genitalia. If the amount of vaginal discharge increases, or its odor changes due to vaginitis, recommend that they consult a primary physician or visit a gynecologist to be examined.<br>5) As the functions of the pelvic floor muscles decline due to decreased levels of female hormones, teach them exercises to strengthen those muscles. Perform these exercises together if the participants so wish.<br>6) If the participants suffer from sleep disorders, take their distress seriously, and work with them to consider ways of relaxation.<br>7) Hot flashes, if caused by tamoxifen or aromatase inhibitors, usually wear off within a few months following the start of therapy (Breast Cancer Clinical Practice Guidelines, 2018). Nevertheless, consider different ways to counter these symptoms, such as trying and incorporating creative clothing ideas and physical exercises into one's daily life and avoiding eating spicy and/or pungent foods. Work with the participants to consider what they want to do<br>8) Some participants may experience a reduction in sexual desire due to lowered estrogen levels. As thoughts on sex are unique to each person, adopt a sympathetic and accepting approach, while hearing their views and attitudes toward sex. Accept the fact that they will experience diverse emotions and maintain an empathetic attitude. Examples of emotions include: while experiencing breast cancer, they feel sorry for their partners; conflicting feelings of both wanting to have sex and not wanting to have sex; and a feeling of wanting to cherish and feel physical affection other than sex. Tell them that they can make decisions based on their own wishes.<br>9) Regard the fact that sets of value towards sex can vary due to changes in marital relationships and aging. Sexual activity/intercourse is not only about the physical aspects. Work	**1. Understand sexual health care needs**<br>1) Based on the interactions, identify the aspects about each patient's sexuality that they cherish, and the things they regard as most important. Use the assessment sheet to conduct assessments.<br>**2. Think together about the meaning of taking care of oneself**<br>2) Respect the personal thoughts and feelings of the participants.<br>3) Ask them about the effects of having the mindset of taking care of themselves, carrying out actions to cherish themselves, improving their physical conditions, healing, and pleasing themselves. Ask them what meaning these have, and convey what meaning there is to them.<br>4) Ask them about the times and occasions they are aware of their desires as a woman and affirm their answers. Describe their desires as seen by a midwife.<br>5) If actions and choices are observed for undergoing routine medical checkups exams and conducting breast self-examinations, convey that they are cherishing their own priceless body and mind, and offer words of praise, approval, and acceptance. Even if the participants fail to sufficiently carry out actions such as undergoing routine medical checkups and exams, and conducting breast self-examinations, affirm their hard work up to now, interact with them with the attitude of waiting for actions and choices, and convey the advantages to women who carry out such actions and choices.

	their feelings and mindsets.<br>8) Maintain an attitude of respecting the participants as human beings who are understanding that their emotions and experiences have meanings, while trying to learn about them.		with breast cancer survivors to consider the meaning of sexual intercourse and its ideal forms for them.<br>10) Ask them if their relationship with their partners has changed since undergoing breast cancer surgery. If positive emotions are expressed regarding their relationship with their partner, accept them with an empathetic and affirmative attitude. If negative emotions are expressed, listen to what they say while accepting, and not dismissing them. While conveying thankfulness for their sharing their story, maintain a feeling of closeness, based on the attitude of working and thinking together<br>11) While undergoing endocrine therapy, the recipients are in a state in which they cannot conceive, as menstruation stops due to the influence of estrogen. Depending on the age of onset, some women may have to give up hope of getting pregnant and giving birth. When the participants talk about their thoughts on pregnancy and giving birth, listen attentively and thoroughly. Respect the fact that they have made the decision to live, and that they are currently undergoing endocrine therapy.	
**Examples of the experiences that have been brought about by care**	The experience of having access to “a place where I can talk as much as I want about myself, in a body that has lost its breasts and fertility and has been damaged,” and the experience of “Sharing with a midwife who accepts ‘me’ as I am, ” the experience of “Turning the gaze on the entangled body and recovering the voice of the body,” and the experience of “Discovering a new meaning of one's existence through living in a body that cannot give birth, a body that can give birth, and a woman's body.”			
