# Supplementary material for: Early-Stage Evaluation of a Novel Sexual Health Care Model for Breast Cancer Survivors Undergoing Endocrine Therapy: A Case Series
Source: Nurs Rep. 2026 Sep 6;16(9):324. doi: 10.3390/nursrep16090324 (PMC13610420; doi:10.3390/nursrep16090324)
Supplement: Supplementary file 1 [file nursrep-16-00324-s001.zip › Table S2.pdf]

Supplementary Table S2. Individual pre- and post-care Self-affirmation Scale Ver. 2 scores and change values.

	**Pre-care score**	**Post-care score**	**Change**
Participant A	18	22	+4
Participant B	25	29	+4
Participant C	27	31	+4
Participant D	15	20	+5
Participant E	17	24	+7
